# Consumption and direct costs of dental care for patients with head and neck cancer: A 16-year cohort study

**DOI:** 10.1371/journal.pone.0182877

**Published:** 2017-08-23

**Authors:** Duangjai Lexomboon, Pär Karlsson, Jan Adolfsson, Anders Ekbom, Aron Naimi-Akbar, Shahram Bahmanyar, Scott Montgomery, Gunilla Sandborgh-Englund

**Affiliations:** 1 Academic Centre for Geriatric Dentistry, Stockholm, Sweden; 2 Department of Health Sciences, Karlstad University, Karlstad, Sweden; 3 Centre for Pharmacoepidemiology, Department of Medicine, Karolinska Institutet, Stockholm, Sweden; 4 Department of Clinical Sciences, Interventions and Technology, Karolinska Institutet, Stockholm, Sweden; 5 Clinical Epidemiology Unit, Department of Medicine, Karolinska Institutet, Stockholm, Sweden; 6 Department of Dental Medicine, Karolinska Institutet, Stockholm, Sweden; 7 Clinical Epidemiology and Biostatistics, School of Medical Sciences, Örebro University, Örebro, Sweden; 8 Department of Epidemiology and Public Health, University College London, London, United Kingdom; International University of Health and Welfare School of Medicine, JAPAN

## Abstract

Patients with head and neck (H&N) cancer are commonly treated with surgery and/or radiotherapy, which can increase the risk of oral infection, dental caries, and periodontal disease. The present study investigated dental care consumption and costs in patient with H&N cancer before and after the cancer diagnosis. Data from Swedish regional and national registers were used to follow up dental care utilization and dental procedure costs. The analysis included 2,754 patients who had been diagnosed with H&N cancer (exposed cohort) in Stockholm County, Sweden, during 2000–2012 and 13,036 matched persons without cancer (unexposed cohort). The exposed cohort was sub-grouped into irradiated and non-irradiated patients for analysis. The exposed cohort underwent a moderately higher number of dental procedures per year than the unexposed cohort in both the year of the cancer diagnosis and the year after cancer diagnosis; in addition, these numbers were higher in the irradiated than in the non-irradiated subgroup of the exposed cohort. Dental care consumption and costs in the exposed cohort declined over time but remained at a slightly higher level than in the unexposed cohort over the long term (more than two years). Examinations and preventive procedures accounted for most of the higher consumption in the short term (2 years) and at the longer term follow-up. Swedish national insurance subsidized costs for dental treatment, which were highest in the irradiated subgroup and lowest in the unexposed cohort. Direct costs to the patient, however, were similar among the groups. Swedish national health insurance protects patients with H&N cancer from high dental expenditures. Further studies on the cost-effectiveness of preventive dental care for patients are needed.

## Introduction

Head and neck (H&N) cancer includes cancers of the oral cavity, sinus & nasal cavities, pharynx, and larynx. It is estimated that 686,000 new diagnoses of H&N cancer were made worldwide in 2012 [1]. Prevalence is higher in men, and in men and women at age 50+ years [2]; major risk factors are alcohol, tobacco and exposure to the human papilloma virus [3–6].

Most H&N cancers are squamous cell in origin. Less common cell origins include salivary gland cells, lymphoid cells, and metastasized cells from distant tumours [7].

Treatment usually comprises surgery and/or radiation therapy, based on the stage, type and site of the cancer, the patient’s condition, and the expected functional outcome [8]. Treatment may also include chemotherapy, as combined or concurrent therapy. Attempts to assure total cancer removal often necessitate surgical margins that are mutilating, requiring the removal of large masses of tissue or entire organs. Subsequent facial and oral reconstruction is then necessary to restore functions and improve the esthetics and quality of life.

Surgical excision and resection can involve major and large areas of the minor salivary glands. Salivary glands are also highly sensitive to radiation [9]; consequently, irradiated patients often present with reduced salivary production. Acute radiation effects are caused by acinar cell atrophy and cell death, which occur within a few days or weeks after radiation treatment. Chronic radiation effects are caused by damage to the connective tissue and epithelium of the gland, blood vessels, and nerves within the gland [10]. Change in salivary secretion and salivary composition can lead to severe and progressive tooth decay, chronic periodontitis, and oral mucosal discomfort [11].

Before cancer treatment, any needed dental treatment and preventive dental care should be done in order to reduce post-treatment risk of infection and dental complications [12, 13]. Teeth with doubtful prognoses should be extracted due to the risk of osteoradionecrosis when extractions are done after radiation therapy. Oral mucositis is a common side effect of radiation in the treatment of H&N cancer and requires attention and supportive care. Dental treatment needs will thus be higher in cancer patients, both during pre-treatment and later as a consequence of the cancer treatment.

In Sweden, various subsidies and schemes within the national health insurance system cover dental care [14]. Swedish county councils are responsible for ensuring that all residents are able to receive the dental care they needed. In 2015, the councils spent about 2.3% of the total net cost for health care on dental care [15]. To our knowledge, no study to date has explored the cost of dental care for H&N cancer patients. The objective of the present study was to investigate the short- and long-term consumption and costs of dental care in patients with H&N cancer.

## Materials and methods

This cohort study is based on data from regional and national Swedish registers. The personal identification number (PIN), which has been assigned to each Swedish resident since 1947, made it possible to link patient registry data with individual records [16]. The study population comprised two cohorts: one exposed cohort and one matched unexposed cohort.

### Data sources

The Swedish Head and Neck Cancer Register for Oral Cancer (SweHNCR) [17] contains information such as diagnosis, cancer stage, treatment plan, and treatment received as well as data on relapses and survival. Patients who were identified through SweHNCR as diagnosed with H&N cancer made up the exposed cohort. All incident diagnoses were in persons living in Stockholm County who had been diagnosed between January 1999 and December 2013 with H&N cancer (International Classification of Diseases [ICD] version 2010, codes ICD9 140–149 and 160–161; ICD10 C00–C14 and C30–C32).

Statistics Sweden maintain national population registers that contain demographic information, such as birthdate, sex, educational level, birth parish, (the Total Population Register) [18], migration (the Register of Population and Population Changes) [18], and other socio-demographic factors such as income, social welfare benefits, and educational level (the Longitudinal integration database for health insurance and labour market studies [LISA] [19]. One of the population registers maintained by the Swedish National Board of Health and Welfare is the Cause of Death Register [20], which records the date and the primary and the contributory causes of death, coded according to ICD.

By linking the Total Population Register with the SweHNCR, Statistics Sweden was able to construct an unexposed cohort that was matched in all characteristics but cancer with the unexposed cohort. Statistics Sweden matched five Stockholm residents with each exposed patient for sex, age, and county of residency in the year before diagnosis. The unexposed cohort members were alive and residents of Stockholm on the date the matched patients were exposed (diagnosed).

The Swedish Social Insurance Agency (SSIA) maintains data on general dental treatments, including treatment and diagnostic codes and procedure cost. SSIA reports this information, which is used for reimbursement purposes [21], to the Dental Health Register at the National Board of Health and Welfare [22]. Both registers were started in July 2008.

Since the National Dental Health Insurance scheme was reformed in 1999, each county council administers and subsidizes dental care that is part of medical healthcare treatment: dental healthcare treatment (DHT). DHT is not reported to the SSIA or the Dental Health Register.

### Demographics

The total Population Register, the Register of Population and Population Changes, and the Cause of Death Register were used to identify dates of birth, death, immigration, and emigration for all subjects. Information on socio-economic characteristics including educational level and income, was obtained through linkage to LISA. The level of education was the highest education attained, categorized into primary school, secondary school, and post-secondary school (university level). Income data were retrieved as family derived individual income, that is, the sum of the income of all family members divided by the number of members.

### Dental care

SSIA supplied information on dental care consumption, while Stockholm County’s administrative register provided data on DHT. Thus, date of treatment, the procedure codes, and dental care costs were retrieved from SSIA. Number of remaining teeth at baseline was obtained from the Dental Health Register. Data from SSIA and Dental Health Register were retrieved from July 2008 and December 2013. DHT data, which included year of treatment, procedure codes, and dental care costs, were retrieved between 1999 and December 2013.

Total annual dental care consumption was obtained by summing data from SSIA and DHT. Dental procedures were grouped according to the procedure codes in the Dental Care Benefits Scheme (Table 1).

**Table 1:** Categories and dental care procedures.

Category	Description
Examination	All procedures related to the clinical examination, including clinical and radiographic examination, saliva sampling, biopsies
Preventive and supportive procedures	Information and non-operative procedures. Non-surgical treatment of periodontal disease, caries excavation, and temporary fillings
Restorative procedures	Permanent fillings
Prosthodontic procedures	All procedures related to fixed, removable and implant supported prosthodontics
Surgical procedures (dento-alveolar)	Tooth extractions, implant placement, periodontal surgery
Endodontic procedures	All procedures related to endodontics

### Exposure

The exposure date was defined as the date of the cancer diagnosis. The exposed cohort was dichotomized into patient with H&N cancer who had received radiotherapy and patient with H&N cancer who did not receive radiotherapy.

### Outcome variables

The two outcome variables are annual costs for dental care (direct costs to the patient and total costs) and annual number of dental procedures.

In the analyses of long-term effects, annual costs were the mean cost or the mean number of procedures per year, where the mean was calculated for each patient over the available years (2009–2013).

### Follow-up period

The cohort members were followed in person-years from the entry date until the date of death or migration, or until the end of the follow-up (31 December 31 2013), whichever occurred first. If the patient died or migrated before the end of the follow-up, the matched subjects in the unexposed cohort were removed from the analysis at that time point.

Dental care consumption and cost were compared between the exposed irradiated cohort, the exposed non-irradiated cohort, and the unexposed cohort. Two follow-ups were done:

- The short-term follow-up comprised data from two years before and after the date of cancer diagnosis.
- The long-term follow-up, comprised data in the year after the short-term follow-up (beyond the first two calendar years after cancer diagnosis).

For a year to be included in the analyses, a patient had to be followed for the complete calendar year.

### Statistical analysis

The dental care consumption, both costs and number of procedures per year, was analyzed by analysis of covariance (ANCOVA). The models included the following categorical covariates: subgroup (exposed non-irradiated subgroup, exposed irradiated subgroup, and unexposed cohort), year of diagnosis (index year), year relative to index year, age (<20, 20–59, 60–79, 80+ years), number of teeth at baseline (0–9, 10–19, 20–32, missing information), education (primary school, secondary school, university, missing information), and income based on tertiles (lower 3^rd^, middle 3^rd^, upper 3^rd^, missing information). Age, number of teeth at baseline, educational level, and income were recorded for the index year.

In the short-term analysis, subjects are random effects. In the long-term analysis, the “group*year” interaction was not statistically significant. The subjects and the covariate “year relative to index year” were excluded from the long-term follow-up model since annual number of procedures or costs were means over the available year (2009–2013). Number of remaining teeth was not recorded prior to 2008, so this variable was also not included in the ANCOVA models of the long-term follow-up.

All analyses were done using the Statistical Analysis System (SAS®) package version 9.4. The Regional Ethical Review Board in Stockholm, Sweden, approved the study (Dnr 2012/2051-31/5 and 2015/257-32).

## Results

In total, 2,754 persons were included in the exposed cohort and 13,036 persons in the unexposed. One person in the exposed cohort had no matched unexposed persons and was excluded; the overall matching ratio was 4.7.

The short-term follow-up included patients with H&N cancer who were diagnosed between January 2010 and December 2012 and for whom data on dental treatment between 2009 and 2013 were available, and the matched unexposed cohort. Thus, the analysis comprised 834 in the exposed cohort and 4,117 in the unexposed (Table 2). In the exposed cohort, 38% had received radiation treatment.

**Table 2:** Number of individuals in each cohort in relation to study design.

Category	Exposed cohort			Unexposed cohort
	Total	Non irradiated	Irradiated	
From registry	2 754	1 422	1 332	13 036
Matched	2 753	1 421	1 332	13 036
Short-term follow-up^1^	834	316	518	4 117
Long-term follow-up^2^	1 032	640	392	4 622

^1^ Follow-up for 2 years before and after cancer diagnosis.

^2^ Follow-up > 2 years after cancer diagnosis.

The long-term follow-up comprised patients diagnosed with H&N cancer between January 2000 and December 2007 for whom data on dental treatment during 2009–2013 were available, and the matched unexposed cohort. The total number was 1,032 persons in the exposed cohort and 4,662 in the unexposed. Among the exposed cohort, 38% received radiation treatment.

### Short-term follow-up

Table 3 presents the demographic characteristics of the two subgroups in the exposed cohort and of the unexposed cohort. The exposed cohort had a slightly lower level of education and income compared to the unexposed. They also had a slightly lower number of remaining teeth at baseline. The irradiated subgroup contained a lower proportion of females compared to the non-irradiated subgroup. In addition, the irradiated subgroup were younger, consumed less dental care and more often had no dental records prior to cancer diagnosis compared to the non-irradiated subgroup and the unexposed cohort.

**Table 3:** Characteristics of the exposed and unexposed cohorts in the short-term follow-up^1^.

Characteristics^2^	Description	Exposed cohort			Unexposed
		Total	Non-irrad	Irrad	cohort
Total	N	834 (100.0%)	316 (100.0%)	518 (100.0%)	4117 (100.0%)
Sex	Male	500 (60.0%)	161 (50.9%)	339 (65.4%)	2465 (59.9%)
	Female	334 (40.0%)	155 (49.1%)	179 (34.6%)	1652 (40.1%)
Age (years)	Mean (SD)	65.7 (14.6)	67.7 (15.2)	64.5 (14.1)	65.5 (14.5)
	< 20	8 (1.0%)	3 (0.9%)	5 (1.0%)	42 (1.0%)
	20 –< 60	240 (28.8%)	73 (23.1%)	167 (32.2%)	1206 (29.3%)
	60 –< 80	456 (54.7%)	178 (56.3%)	278 (53.7%)	2252 (54.7%)
	80 +	130 (15.6%)	62 (19.6%)	68 (13.1%)	617 (15.0%)
Education	Missing	20 (2.4%)	11 (3.5%)	9 (1.7%)	90 (2.2%)
	Primary	214 (25.7%)	86 (27.2%)	128 (24.7%)	995 (24.2%)
	Secondary	351 (42.1%)	122 (38.6%)	229 (44.2%)	1634 (39.7%)
	University	249 (29.9%)	97 (30.7%)	152 (29.3%)	1398 (34.0%)
Family income	Missing	4 (0.5%)	1 (0.3%)	3 (0.6%)	10 (0.2%)
	Lower 3rd	196 (23.5%)	76 (24.1%)	120 (23.2%)	832 (20.2%)
	Middle 3rd	267 (32.0%)	106 (33.5%)	161 (31.1%)	1298 (31.5%)
	Upper 3rd	367 (44.0%)	133 (42.1%)	234 (45.2%)	1977 (48.0%)
Number of teeth	Mean (SD)	24.2 (7.2)	24.0 (7.4)	24.3 (7.1)	24.8 (6.3)
	20 or more	489 (58.6%)	197 (62.3%)	292 (56.4%)	2721 (66.1%)
	10–19	73 (8.8%)	28 (8.9%)	45 (8.7%)	312 (7.6%)
	0–9	42 (5.0%)	18 (5.7%)	24 (4.6%)	149 (3.6%)
	Missing	230 (27.6%)	73 (23.1%)	157 (30.3%)	935 (22.7%)
Costs^3^	Mean (SD)	3440 (8007)	3526 (6738)	3387 (8699)	3546 (7423)
Any dental procedure^4^	N	501 (60.6%)	212 (67.5%)	289 (56.3%)	2808 (68.5%)

^1^ Comprises patients who were diagnosed with H&N cancer during 2010–2012, had recorded data in the time period 2009–2013 and recorded data for the time frame ±2 years of the year of cancer diagnosis, and the unexposed cohort.

^2^ All characteristics determined at baseline, the year before the year of cancer diagnosis.

^3^ Total annual costs for dental care utilization (SEK 100 is approximately EUR 10).

^4^ Number of subjects with at least one dental procedure on record

Non-irrad = Non-irradiated; Irrad = Irradiation; SD = standard deviation

**Fig 1:**
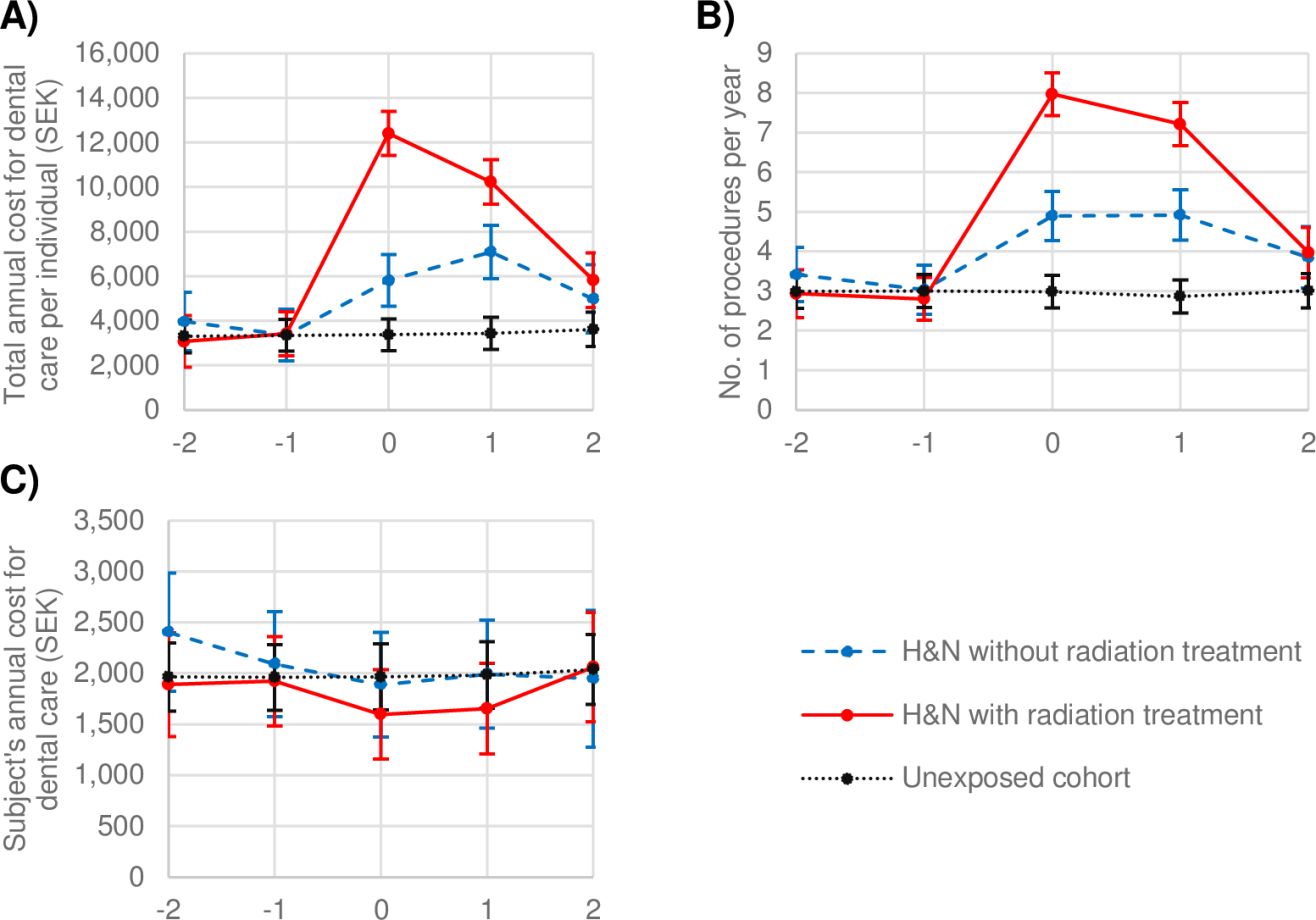
Short-term follow-up of the exposed subgroups with and without radiation treatment, and the unexposed cohort for two years before and after the diagnosis of cancer. **ANCOVA model results.** A) Mean total cost for dental care. B) Mean number of annual dental procedures. C) Mean direct costs to the patient.

Dental care consumption increased significantly in conjunction with a diagnosis of H&N cancer. Fig 1 shows the adjusted mean dental care consumption two years before and after a diagnosis of cancer. In the year of diagnosis and in the following year, total costs were statistically significantly higher in the exposed cohort (Fig 1A); the cohort also underwent a higher number of dental procedures (Fig 1B). The irradiated subgroup had statistically significantly higher costs and numbers of procedures compared to the unexposed cohort and the non-irradiated subgroup (ANCOVA, *p* < .0001). Fig 1C shows, direct costs to the patient did not differ significantly between groups.

Table 4 shows the differences between cohorts for dental procedure categories. The main difference was a statistically significantly higher number of examinations and of preventive and supportive procedures. The irradiated subgroup had a mean of 3.9 examination procedures in the year of diagnosis, whereas the corresponding number in non-irradiated patients with H&N cancer was 2.1. The numbers of examination procedures declined for the two following years but remained higher than in the unexposed cohort. The unexposed cohort remained at a relatively stable level of 1.1–1.2 procedures per year. The same pattern was seen for preventive and supportive procedures, that is, a considerable increase in the irradiated subgroup, from 0.7 procedures per year before treatment up to 2.7 the year after the diagnosis of cancer. The same pattern was observed, but less pronounced, in the non-irradiated subgroup. In addition, numbers of prosthodontic-related and surgical procedures increased in both subgroups.

**Table 4:** Short-term follow-up: Dental treatment before and after cancer diagnosis. Numbers of dental procedures annually in the exposed and unexposed cohorts (ANCOVA model)^1^.

Dental procedure category	Year of relative diagnosis	Exposed cohort		Unexposed	Pairwise comparisons		
		Non-irradiated	Irradiated	cohort	*(p-value)*		
		LSMeans	LSMeans	LSMeans	Non-irrad vs Unexp	Irrad vs Unexp	Non-irrad vs Irrad
		(95% CI)	(95% CI)	(95% CI)			
Examination	-2	1.05 (0.79–1.31)	1.01 (0.78–1.24)	1.08 (0.92–1.24)	NS	NS	NS
	-1	1.16 (0.93–1.40)	0.98 (0.77–1.18)	1.10 (0.94–1.26)	NS	NS	NS
	0	2.10 (1.86–2.34)	3.88 (3.67–4.08)	1.12 (0.96–1.28)	< .001	< .001	< .001
	1	1.89 (1.65–2.13)	2.88 (2.68–3.09)	1.10 (0.95–1.26)	< .001	< .001	< .001
	2	1.46 (1.16–1.76)	1.43 (1.19–1.67)	1.19 (1.03–1.36)	NS	< .05	NS
Preventive and supportive	-2	0.81 (0.60–1.03)	0.74 (0.55–0.93)	0.73 (0.59–0.87)	NS	NS	NS
	-1	0.79 (0.59–0.99)	0.68 (0.51–0.85)	0.73 (0.60–0.87)	NS	NS	NS
	0	1.36 (1.16–1.55)	2.48 (2.31–2.66)	0.75 (0.61–0.89)	< .001	< .001	< .001
	1	1.65 (1.45–1.85)	2.67 (2.49–2.84)	0.72 (0.58–0.86)	< .001	< .001	< .001
	2	1.10 (0.86–1.33)	1.20 (1.00–1.39)	0.74 (0.59–0.88)	< .001	< .001	NS
Restorative	-2	0.70 (0.49–0.90)	0.43 (0.25–0.62)	0.44 (0.32–0.57)	< .001	NS	< .05
	-1	0.42 (0.23–0.60)	0.37 (0.21–0.53)	0.46 (0.34–0.58)	NS	NS	NS
	0	0.55 (0.36–0.73)	0.28 (0.12–0.44)	0.44 (0.32–0.56)	NS	< .01	< .01
	1	0.40 (0.21–0.59)	0.32 (0.16–0.49)	0.38 (0.26–0.51)	NS	NS	NS
	2	0.41 (0.17–0.64)	0.46 (0.27–0.65)	0.40 (0.27–0.53)	NS	NS	NS
Prosthodontic	-2	0.58 (0.36–0.80)	0.48 (0.28–0.67)	0.46 (0.34–0.58)	NS	NS	NS
	-1	0.43 (0.24–0.63)	0.41 (0.25–0.58)	0.44 (0.32–0.55)	NS	NS	NS
	0	0.49 (0.30–0.68)	0.67 (0.51–0.84)	0.41 (0.29–0.52)	NS	< .001	NS
	1	0.66 (0.46–0.86)	0.88 (0.71–1.04)	0.42 (0.31–0.54)	< .01	< .001	< .05
	2	0.56 (0.30–0.82)	0.50 (0.29–0.70)	0.43 (0.31–0.55)	NS	NS	NS
Surgical	-2	0.20 (0.09–0.31)	0.24 (0.15–0.33)	0.20 (0.15–0.26)	NS	NS	NS
	-1	0.18 (0.09–0.28)	0.30 (0.22–0.38)	0.20 (0.15–0.26)	NS	< .01	< .05
	0	0.32 (0.22–0.41)	0.47 (0.40–0.55)	0.18 (0.13–0.24)	< .01	< .001	< .01
	1	0.20 (0.11–0.30)	0.25 (0.17–0.33)	0.17 (0.12–0.23)	NS	< .05	NS
	2	0.16 (0.03–0.28)	0.26 (0.16–0.36)	0.17 (0.11–0.23)	NS	< .05	NS
Endodontic	-2	0.06 (-.00–0.12)	0.03 (-0.03–0.08)	0.07 (0.03–0.10)	NS	NS	NS
	-1	0.03 (-.02–0.09)	0.06 (0.01–0.10)	0.06 (0.03–0.09)	NS	NS	NS
	0	0.04 (-.01–0.09)	0.07 (0.03–0.12)	0.07 (0.04–0.10)	NS	NS	NS
	1	0.03 (-.02–0.09)	0.06 (0.01–0.10)	0.06 (0.03–0.09)	NS	NS	NS
	2	0.11 (0.03–0.18)	0.04 (-0.01–0.10)	0.07 (0.04–0.10)	NS	NS	NS

^1^ The ANCOVA model included covariates described in Table 3: Group (Exposed non-irradiated subgroup, exposed irradiated subgroup, and unexposed cohort), year of diagnosis (index year), year relative to the year of cancer diagnosis, age, oral status (number of teeth), highest education, family derived individual income.

NS = not significant, Non-irrad = non-irradiated; Irrad = Irradiated, Unexp = unexposed; LSMeans = Least-squares means

### Long-term follow-up

Table 5 presents the characteristics of the persons included in the analysis. The two cohorts had similar age and sex distributions. Similar to persons included in the short-term follow-up, the unexposed cohorts had slightly higher education and income than the exposed cohort. The mean age at diagnosis in the long-term follow-up was lower compared to the cohorts in the short-term follow-up (Tables 3 and 5).

**Table 5:** Characteristics of the exposed and unexposed cohorts in the long-term follow-up^1^.

Characteristic^2^	Description	Exposed cohort			Unexposed
		Total	Non-irradiated	Irradiated	cohort
Total	N	1032 (100.0%)	640 (100.0%)	392 (100.0%)	4622 (100.0%)
Sex	Male	623 (60.4%)	369 (57.7%)	254 (64.8%)	2765 (59.8%)
	Female	409 (39.6%)	271 (42.3%)	138 (35.2%)	1857 (40.2%)
Age (years)	Mean (SD)	61.7 (13.5)	62.7 (14.0)	60.0 (12.5)	60.6 (13.1)
	< 20	9 (0.9%)	8 (1.3%)	1 (0.3%)	43 (0.9%)
	20 –< 60	433 (42.0%)	249 (38.9%)	184 (46.9%)	2089 (45.2%)
	60 –< 80	496 (48.1%)	311 (48.6%)	185 (47.2%)	2189 (47.4%)
	80 +	94 (9.1%)	72 (11.3%)	22 (5.6%)	301 (6.5%)
Education	Missing	24 (2.3%)	19 (3.0%)	5 (1.3%)	107 (2.3%)
	Primary	299 (29.0%)	191 (29.8%)	108 (27.6%)	1147 (24.8%)
	Secondary	421 (40.8%)	256 (40.0%)	165 (42.1%)	1905 (41.2%)
	University	288 (27.9%)	174 (27.2%)	114 (29.1%)	1463 (31.7%)
Family income	Missing	7 (0.7%)	7 (1.1%)		35 (0.8%)
	Lower 3rd	382 (37.0%)	237 (37.0%)	145 (37.0%)	1608 (34.8%)
	Middle 3rd	372 (36.0%)	230 (35.9%)	142 (36.2%)	1556 (33.7%)
	Upper 3rd	271 (26.3%)	166 (25.9%)	105 (26.8%)	1423 (30.8%)

^1^ Includes patients who were diagnosed with H&N cancer during 2000–2007 and had recorded data in the time period 2009–2013, and the unexposed cohort.

^2^ All characteristics determined at baseline, the year before the year of cancer diagnosis.

SD = standard deviation

Tables 6 and 7 present the ANCOVA results for the adjusted total dental care consumption. Total dental care costs were statistically significantly higher in the exposed cohort compared to the unexposed cohort (*p* < .0001), whereas direct costs to the patient were significantly lower in the irradiated subgroup, compared to the non-irradiated and to the unexposed cohort. The exposed cohort received a significantly higher number of procedures in all procedure categories except for endodontics (Table 7). Restorative procedures were significantly higher in the irradiated subgroup compared to the unexposed cohort (*p* < .05), but no significant difference between non-irradiated subgroup and the unexposed cohort.

**Table 6:** Long-term follow-up: Dental treatment costs (direct costs to the patient and total costs) in the exposed and unexposed cohorts (ANCOVA model)^1^.

Variable	Exposed subgroup		Unexposed	Pairwise comparisons		
	Non-irradiated	Irradiated	cohort	(*p-value*)		
	LSMeans	LSMeans	LSMeans	Non-irrad vs Unexp	Irrad vs Unexp	Non-irrad vs Irrad
	(95% CI)	(95% CI)	(95% CI)			
Direct costs to the patient	1578 (1312–1844)	1205 (882–1528)	1551 (1352–1750)	NS	< .05	< .05
Total costs	4673 (4065–5282)	4730 (3992–5468)	2572 (2116–3027)	< .001	< .001	NS

^1^ The ANCOVA model included covariates as described in Table 5: Group (Exposed non-irradiated subgroup, Exposed irradiated subgroup, and Unexposed cohort), year of diagnosis (index year), age, highest education, family derived individual income costs in SEK (SEK 100 is approximately EUR 10)

NS = not significant; Non-irrad = non-irradiated; Irrad = irradiated; Unexp = unexposed; LSMeans = Least-squares means

**Table 7:** Long-term follow-up: Number of annual dental procedures in the exposed and unexposed cohorts (ANCOVA model)^1^.

Dental care procedure category	Exposed subgroup		Unexposed	Pairwise comparisons		
	Non-irradiated	Irradiated	cohort	(*p-value*)		
	LSMeans	LSMeans	LSMeans	Non-irrad vs Unexp	Irrad vs Unexp	Non-irrad vs Irrad
	(95% CI)	(95% CI)	(95% CI)			
All dental procedures	3.51 (3.15–3.87)	3.44 (3.00–3.87)	2.70 (2.43–2.97)	< .001	< .001	NS
Examination	1.38 (1.25–1.51)	1.28 (1.12–1.44)	1.11 (1.01–1.21)	< .001	< .05	NS
Preventive and supportive	0.88 (0.76–0.99)	0.93 (0.79–1.06)	0.73 (0.64–0.81)	< .001	< .001	NS
Restorative	0.54 (0.43–0.64)	0.61 (0.48–0.74)	0.48 (0.40–0.55)	NS	< .05	NS
Prosthodontic	0.45 (0.35–0.54)	0.34 (0.23–0.46)	0.21 (0.14–0.29)	< .001	< .01	NS
Surgical	0.18 (0.14–0.23)	0.18 (0.12–0.23)	0.11 (0.08–0.14)	< .001	< .01	NS
Endodontic	0.05 (0.03–0.08)	0.05 (0.02–0.08)	0.05 (0.04–0.07)	NS	NS	NS

^1^ The ANCOVA model included covariates as described in Table 5: Group (Exposed non-irradiated subgroup, Exposed irradiated subgroup, and Unexposed cohort), year of diagnosis (index year), age, highest education, family derived individual income costs in SEK (SEK 100 is approximately EUR 10)

NS = not significant; Non-irrad = non-irradiated; Irrad = irradiated; Unexp = unexposed; LSMeans = Least-squares means

## Discussion

The present study uses register data on the Stockholm County population from 1999 to 2013 to compare dental treatment consumption and costs in a period of two years before and after a cancer diagnosis and more than two years after a cancer diagnosis, between patients with H&N cancer and patients without H&N cancer, matched by age and sex. Sub-group comparisons were also made between the unexposed cohort, patients with H&N cancer who had received radiation therapy, and patients with H&N cancer who did not. The results show a statistically and clinically significant increase in consumption and costs for dental care among irradiated and non-irradiated subgroups in the year of diagnosis and the year after. Consumption and costs for dental care decline slowly but remain slightly higher compared to the unexposed cohort, also in the long-term follow-up.

Date of cancer diagnosis and the unavailability of data on confounding factors may affect the results. The populations of patients with H&N cancer in the short- and long-term follow-ups were diagnosed during different time periods and may have received varying health and dental care as well as financial support. Between 1999 and 2013, however, there was no major change in the dental care remuneration system [14], and the influence of the year of cancer diagnosis on the comparison between the short- and long-term follow-ups should be minimal.

Confounding factors might influence the between-group comparisons. First, data on smoking and alcohol were unavailable. Tobacco and alcohol consumption have been shown to increase the risk of oral and oropharyngeal squamous cell carcinoma in a Swedish population [23, 24]; in addition, these risk factors are also associated with tooth loss [25, 26]. However, the mean number of teeth and the annual number of dental procedures at one year and two years before cancer diagnosis were similar, suggesting that the cohorts had similar dental health prior to the cancer diagnosis.

Second, the unexposed cohorts in both the short- and long-term follow-ups were more highly educated and had higher incomes, both of which have been shown to be positively associated with dental care consumption [27, 28]. In contrast, dental care utilization and costs for dental care before cancer diagnosis were similar, which indicates that lower income and education should not contribute significantly to the differences in dental care consumption and cost for care in the year of the cancer diagnosis and the follow-up years in this study. Therefore, time of cancer diagnosis, smoking, and alcohol were not expected to substantially influence the between-group comparisons.

The strength of the study is the completeness of the data from the various registries linked through the Swedish PIN, including all patients diagnosed with H&N cancer in Stockholm County. The missing data after matching were only due to death or emigration.

Data on dental care consumption from SSIA were available since July 2008, which restricted the analysis to the present study design. In addition, since the County-administered DHT was not reported to the national registries, the present study is based on patients from Stockholm County only. Thus, the results are not generalizable to other populations.

Dental care utilisation is expected to increase after a diagnosis of H&N cancer, especially in patients who receive radiation treatment [29]. The results of our study also show that the mean numbers of dental procedures and costs were statistically significantly highest in the irradiated subgroup in the year of diagnosis and in the following year.

Considering the types of dental procedures, the differences for procedures related to prevention and support are both statistically and clinically significant. The mean number of annual examinations and preventive procedures in the irradiated subgroup one-year following the cancer diagnosis were 2.9 and 2.7 compared to 1.1 and 0.7 in the unexposed cohort, a difference of about two procedures per year. Short recall intervals are often needed, for example, due to the acute side effects of irradiation such as oral mucositis, a severe inflammation of oral mucosa [30].

Low cariogenic food, and fluoride supplementation can reduce caries development. A study in Australia shows that irradiated patients with H&N cancer who had regular dental examination and recurrent oral hygiene instruction, and patients who adhered strictly to a non-cariogenic diet or used high concentration fluoride toothpaste daily had significantly lower caries development at 12-months post-treatment than non-compliant patients [31]. Thus, rigorous dental procedures related to primary and secondary prevention in the year of diagnosis and the following years should be beneficial in the long term. In the present study, the differences between all types of procedures between the exposed and the unexposed cohorts more than two years following the cancer diagnosis, though statistically significant, are less than 1 procedure per year. Clinically, these annual differences can be considered low.

Under the Swedish insurance system, DHT subsidies (for dental care as part of the medical healthcare treatment) do not cover permanent restorations for caries treatment. This most likely results in the majority of caries treatment procedures being registered as preventive and supportive procedures [32]; as a consequence, we found no significant differences in numbers of restorative procedures before and after the year of cancer diagnosis. In the long-term follow-up, compared to the unexposed cohort, the annual numbers of restorative procedures were statistically higher in the irradiated subgroup but not in the non-irradiated. Clinically, however, the difference can be considered unexpectedly small.

The long-term follow–up found that the exposed cohort had statistically significantly higher total costs and numbers of procedures than the non-exposed. The difference in total yearly costs is about SEK 2,100 (approximately EUR 210), whereas the difference in number of procedures is less than one per year. Although the costs for dental care were higher, the direct costs to the patient did not differ compared to the unexposed cohort. The interpretation is that the exposed cohort is subject to more complicated and expensive procedures compared to the non-exposed. Under the Swedish dental insurance system, the cost of dental treatment is under a high-cost protection scheme [33], which compensates patients in need of extensive dental rehabilitation. In addition, patients who suffer from hyposalivation due to, for example, radiation treatment are entitled to additional compensation through a special dental care allowance, subsidies for preventive and restorative care, in order to reduce the risk of deteriorating dental health. The results of the present study indicate that the Swedish dental insurance system is efficient in the protection of these vulnerable patient groups against additional expenditure for dental care.

Comparisons of costs for dental treatment in the short- and long-term analyses found that the differences in subsidized total costs between the irradiated cohort and the unexposed cohort in the diagnosis year were about SEK 9,000 (approximately EUR 900) per person and year, but was only about SEK 2,100 (approximately EUR 210) in the long-term follow-up.

## Conclusions

The present study reports a marked increase in consumption and costs for dental care among irradiated and non-irradiated patients with H&N cancer in the year of diagnosis and the following year. Thereafter, dental care consumption and costs decline but remains at a slightly higher level than among subjects with no H&N cancer. Frequent examination and preventive care may protect these patients from additional costs for dental care, but further studies on the cost-effectiveness of dental care for patients with H&N cancer are needed.

## Supporting information

S1 Table Characteristics of the exposed and unexposed cohorts in the short-term follow–up^1^. ^1^ Comprises patients who were diagnosed with H&N cancer during 2010–2012, had recorded data in the time period 2009–2013 and recorded data for the time frame ±2 years of the year of diagnosis, and the matched population without H&N cancer. ^2^ All characteristics determined at baseline, the year before the year of cancer diagnosis. ^3^ Total annual costs for dental care utilization (SEK 100 is approximately EUR 10). ^4^ Number of subjects with at least one dental procedure on record.
(PDF)

S2 Table Characteristics of the exposed and unexposed cohorts in the long-term follow up^1^. ^1^ Comprises patients who were diagnosed with H&N cancer during 2000–2007 and had recorded data in the time period 2009–2013, and the matched population without H&N cancer. ^2^ All characteristics determined at baseline, the year before the year of cancer diagnosis.
(PDF)

S3 Table Short-term follow-up: Costs before and after cancer diagnosis in the exposed and unexposed cohorts—Unadjusted analysis. (PDF)

S4 Table Short-term follow-up: Number of procedures before and after cancer diagnosis in the exposed and unexposed cohorts (ANCOVA model)—Unadjusted analysis. (PDF)

S5 Table Long-term follow-up: Costs before and after cancer diagnosis in the exposed and unexposed cohorts—Unadjusted analysis. (PDF)

S6 Table Long-term follow-up: Number of procedures before and after cancer diagnosis in the exposed and unexposed cohorts (ANCOVA model)—Unadjusted analysis. (PDF)

## References

[1] Ferlay J, Soerjomataram I, Dikshit R, Eser S, Mathers C, Rebelo M, et al Cancer incidence and mortality worldwide: sources, methods and major patterns in GLOBOCAN 2012. Int J Cancer. 2015;136(5): E359–E386. doi: 10.1002/ijc.29210 25220842 10.1002/ijc.29210

[2] Montero PH, Patel SG. Cancer of the oral cavity. Surg Oncol Clin N Am. 2015;24(3): 491–508. doi: 10.1016/j.soc.2015.03.006 25979396 10.1016/j.soc.2015.03.006 PMC5018209

[3] Maasland DH, van den Brandt PA, Kremer B, Goldbohm RA, Schouten LJ. Alcohol consumption, cigarette smoking and the risk of subtypes of head-neck cancer: results from the Netherlands Cohort Study. BMC Cancer. 2014;14: 187 Available from: https://doi.org/10.1186/1471-2407-14-187. 24629046 10.1186/1471-2407-14-187 PMC4004328

[4] Khan Z, Tonnies J, Muller S. Smokeless tobacco and oral cancer in South Asia: a systematic review with meta-analysis. J Cancer Epidemiol. 2014 Available from: http://dx.doi.org/10.1155/2014/394696. 10.1155/2014/394696 PMC4109110 25097551

[5] Zhou J, Michaud DS, Langevin SM, McClean MD, Eliot M, Kelsey KT. Smokeless tobacco and risk of head and neck cancer: evidence from a case-control study in New England. Int J Cancer. 2013;132(8): 1911–1917. doi: 10.1002/ijc.27839 22987222 10.1002/ijc.27839 PMC3552089

[6] Pezzuto F, Buonaguro L, Caponigro F, Ionna F, Starita N, Annunziata C, et al Update on Head and Neck Cancer: Current Knowledge on Epidemiology, Risk Factors, Molecular Features and Novel Therapies. Oncology. 2015;89(3): 125–136. doi: 10.1159/000381717 25967534 10.1159/000381717

[7] Rettig EM, D'Souza G. Epidemiology of head and neck cancer. Surg Oncol Clin N Am. 2015;24(3): 379–396. doi: 10.1016/j.soc.2015.03.001 25979389 10.1016/j.soc.2015.03.001

[8] Cognetti DM, Weber RS, Lai SY. Head and neck cancer: an evolving treatment paradigm. Cancer. 2008;113 Suppl 7: 1911–1932. 18798532 10.1002/cncr.23654 PMC2751600

[9] Grundmann O, Mitchell GC, Limesand KH. Sensitivity of salivary glands to radiation: from animal models to therapies. J Dent Res. 2009;88(10): 894–903. doi: 10.1177/0022034509343143 19783796 10.1177/0022034509343143 PMC2882712

[10] Vissink A, van Luijk P, Langendijk JA, Coppes RP. Current ideas to reduce or salvage radiation damage to salivary glands. Oral diseases. 2015;21(1): e1–e10. doi: 10.1111/odi.12222 24581290 10.1111/odi.12222

[11] Meurman JH, Grönroos L. Oral and dental health care of oral cancer patients: hyposalivation, caries and infections. Oral Oncol. 2010;46(6):464–467. doi: 10.1016/j.oraloncology.2010.02.025 20308007 10.1016/j.oraloncology.2010.02.025

[12] Joshi VK. Dental treatment planning and management for the mouth cancer patient. Oral Oncol. 2010;46(6): 475–9. doi: 10.1016/j.oraloncology.2010.03.010 20400359 10.1016/j.oraloncology.2010.03.010

[13] Jawad H, Hodson NA, Nixon PJ. A review of dental treatment of head and neck cancer patients, before, during and after radiotherapy: part 1. Br Dent J. 2015;218(2): 65–68. doi: 10.1038/sj.bdj.2015.28 25613260 10.1038/sj.bdj.2015.28

[14] Anell A, Glenngard AH, Merkur S. Sweden health system review. Health Syst Transit. 2012;14(5): 1–159. 22894859

[15] Swedish Association of Local Authorities and Regions (SALAR). Statistik om hälso- och sjukvård samt regional utveckling 2015. Swedish. Available from: http://webbutik.skl.se/sv/artiklar/statistik-om-halso-och-sjukvard-samt-regional-utveckling-2015.html.

[16] Ludvigsson JF, Otterblad-Olausson P, Pettersson BU, Ekbom A. The Swedish personal identity number: possibilities and pitfalls in healthcare and medical research. Eur J Epidemiol. 2009;24(11): 659–667. doi: 10.1007/s10654-009-9350-y 19504049 10.1007/s10654-009-9350-y PMC2773709

[17] Regional Cancer Centre. Swedish Head and Neck Cancer Register (SweHNCR). Swedish. Available from: http://www.cancercentrum.se/samverkan/cancerdiagnoser/huvud-och-hals/kvalitetsregister/.

[18] Statistics Sweden. Population. Swedish. Available from: http://www.scb.se/en_/Finding-statistics/Statistics-by-subject-area/Population/.

[19] Statistics Sweden. Longitudinal integration database for health insurance and labor market studies (LISA). Swedish. Available from: http://www.scb.se/lisa/.

[20] The Swedish National Board of Health and Welfare. Cause of Death Register. Swedish. Available from: http://www.socialstyrelsen.se/register/dodsorsaksregistret.

[21] Swedish Social Insurance Agency. Dental care. Swedish. Available from: https://www.forsakringskassan.se/statistik/statistik_och_analys2/statistik_a_o/!ut/p/a1/04_Sj9CPykssy0xPLMnMz0vMAfGjzOJNPFycDd2dDbzdQ9xcDRwdvfyNnY2cDb29DIAKIlEUBAe6GDgahgYZeQSZuQcEmxGn3wAHcDQgpD9cPwpViUFomJuBo6eJn69FYKiRSagJugIsTgQrwOOGgtzQCINMT0UA7GrMqw!!/?1dmy&urile = wcm%3apath%3a%2Fcontentse%2Fstatistik%2Fstatistik%2Fovrigaersatt%2Ftandvard%2Ftandvard.

[22] The Swedish National Board of Health and Welfare. Dental Health Registry. Swedish. Available from: http://www.socialstyrelsen.se/register/halsodataregister/tandhalsoregistret.

[23] Rosenquist K, Wennerberg J, Schildt EB, Bladstrom A, Hansson BG, Andersson G. Use of Swedish moist snuff, smoking and alcohol consumption in the aetiology of oral and oropharyngeal squamous cell carcinoma. A population-based case-control study in southern Sweden. Acta Otolaryngol. 2005;125(9): 991–998. 16193590 10.1080/00016480510043440

[24] Roosaar A, Johansson AL, Sandborgh-Englund G, Axell T, Nyren O. Cancer and mortality among users and nonusers of snus. Int J Cancer. 2008;123(1): 168–173. doi: 10.1002/ijc.23469 18412245 10.1002/ijc.23469

[25] Carson SJ, Burns J. Impact of smoking on tooth loss in adults. Evid Based Dent. 2016;17(3): 73–74. doi: 10.1038/sj.ebd.6401182 27767106 10.1038/sj.ebd.6401182

[26] Heegaard K, Avlund K, Holm-Pedersen P, Hvidtfeldt UA, Bardow A, Gronbaek M. Amount and type of alcohol consumption and missing teeth among community-dwelling older adults: findings from the Copenhagen Oral Health Senior study. J Public Health Dent. 2011;71(4): 318–326. doi: 10.1111/j.1752-7325.2011.00276.x 22320290 10.1111/j.1752-7325.2011.00276.x

[27] Hjern A, Grindefjord M, Sundberg H, Rosen M. Social inequality in oral health and use of dental care in Sweden. Community Dent Oral Epidemiol. 2001;29(3): 167–174. 11409675 10.1034/j.1600-0528.2001.290302.x

[28] Molarius A, Engstrom S, Flink H, Simonsson B, Tegelberg A. Socioeconomic differences in self-rated oral health and dental care utilisation after the dental care reform in 2008 in Sweden. BMC Oral Health. 2014;14: 134 doi: 10.1186/1472-6831-14-134 25403781 10.1186/1472-6831-14-134 PMC4240880

[29] Jensen SB, Pedersen AM, Vissink A, Andersen E, Brown CG, Davies AN, et al A systematic review of salivary gland hypofunction and xerostomia induced by cancer therapies: prevalence, severity and impact on quality of life. Support Care Cancer. 2010;18(8): 1039–1060. doi: 10.1007/s00520-010-0827-8 20237805 10.1007/s00520-010-0827-8

[30] Jawad H, Hodson NA, Nixon PJ. A review of dental treatment of head and neck cancer patients, before, during and after radiotherapy: part 2. Br Dent J. 2015;218(2): 69–74. doi: 10.1038/sj.bdj.2015.29 25613261 10.1038/sj.bdj.2015.29

[31] Frydrych AM, Slack-Smith LM, Parsons R. Compliance of post-radiation therapy head and neck cancer patients with caries preventive protocols. Aust Dent J. 2016 doi: 10.1111/adj.12491 Epub 2016 Nov 18. 27861968 10.1111/adj.12491

[32] The Dental and Pharmaceutical Benefits Agency (TLV). Handbok till TLVFS 2010:2 om statligt tandvårdsstöd version 2.1 Stockholm: Swedish. Available from: www.tlv.se/Global/handbok/101118-handbok-tandvard.pdf.

[33] The Swedish Social Insurance Administration (Försäkringskassan). National dental care subsidy. Swedish. Available from: https://www.forsakringskassan.se/tandvard/statligt_tandvardsstod/!ut/p/z1/04_Sj9CPykssy0xPLMnMz0vMAfIjo8ziLYwMfJ2cDB0NLIINLAw8LT0sXd0sjdy9jM31w8EKDHAARwP9KGL041EQhd_4cP0ovFa4GEEV4DGjIDc0wiDTUREAivSASw!!/dz/d5/L0lDUmlTUSEhL3dHa0FKRnNBLzROV3FpQSEhL2Vu/?keepNavState=true.

