# Supplementary material for: Consumption and direct costs of dental care for patients with head and neck cancer: A 16-year cohort study
Source: PLoS One. 2017 Aug 23;12(8):e0182877. doi: 10.1371/journal.pone.0182877 (PMC5568378; doi:10.1371/journal.pone.0182877)
Supplement: S1 Table — 1 Comprises patients who were diagnosed with H&N cancer during 2010–2012, had recorded data in the time period 2009–2013 and recorded data for the time frame ±2 years of the year of diagnosis, and the matched population without H&N cancer. 2 All characteristics determined at baseline, the year before the year of cancer diagnosis. 3 Total annual costs for dental care utilization (SEK 100 is approximately EUR 10). 4 Number of subjects with at least one dental procedure on record. (PDF) [file pone.0182877.s001.pdf]

S1 Characteristics of the exposed and unexposed cohorts in the short-term follow-up. [1]

Characteristic [2]	Description	Exposed cohort	Unexposed cohort	Exposed: Subgroup Non-irrad	Unexpo matched to Non-irrad	Exposed: Subgroup Irrad	Unexpo matched to Irrad
Total	N	834 (100.0%)	4117 (100.0%)	316 (100.0%)	1571 (100.0%)	518 (100.0%)	2546 (100.0%)
Sex	Male	500 (60.0%)	2465 (59.9%)	161 (50.9%)	798 (50.8%)	339 (65.4%)	1667 (65.5%)
	Female	334 (40.0%)	1652 (40.1%)	155 (49.1%)	773 (49.2%)	179 (34.6%)	879 (34.5%)
Age (years)	Mean (SD)	65.7 (14.6)	65.5 (14.5)	67.7 (15.2)	67.4 (15.1)	64.5 (14.1)	64.3 (14.0)
	<20	8 (1.0%)	42 (1.0%)	3 (0.9%)	17 (1.1%)	5 (1.0%)	25 (1.0%)
	20 ─ <60	240 (28.8%)	1206 (29.3%)	73 (23.1%)	366 (23.3%)	167 (32.2%)	840 (33.0%)
	60 ─ <80	456 (54.7%)	2252 (54.7%)	178 (56.3%)	885 (56.3%)	278 (53.7%)	1367 (53.7%)
	80 +	130 (15.6%)	617 (15.0%)	62 (19.6%)	303 (19.3%)	68 (13.1%)	314 (12.3%)
Education	Missing	20 (2.4%)	90 (2.2%)	11 (3.5%)	34 (2.2%)	9 (1.7%)	56 (2.2%)
	Primary	214 (25.7%)	995 (24.2%)	86 (27.2%)	392 (25.0%)	128 (24.7%)	603 (23.7%)
	Secondary	351 (42.1%)	1634 (39.7%)	122 (38.6%)	629 (40.0%)	229 (44.2%)	1005 (39.5%)
	University	249 (29.9%)	1398 (34.0%)	97 (30.7%)	516 (32.8%)	152 (29.3%)	882 (34.6%)
Family income	Missing	4 (0.5%)	10 (0.2%)	1 (0.3%)	0 (0)	3 (0.6%)	10 (0.4%)
	Lower 3rd	196 (23.5%)	832 (20.2%)	76 (24.1%)	316 (20.1%)	120 (23.2%)	516 (20.3%)
	Middle 3rd	267 (32.0%)	1298 (31.5%)	106 (33.5%)	547 (34.8%)	161 (31.1%)	751 (29.5%)
	Upper 3rd	367 (44.0%)	1977 (48.0%)	133 (42.1%)	708 (45.1%)	234 (45.2%)	1269 (49.8%)
Number of teeth	Mean (SD)	24.2 (7.2)	24.8 (6.3)	24.0 (7.4)	24.3 (6.5)	24.3 (7.1)	25.2 (6.2)
	20 or more	489 (58.6%)	2721 (66.1%)	197 (62.3%)	1019 (64.9%)	292 (56.4%)	1702 (66.8%)
	10─19	73 (8.8%)	312 (7.6%)	28 (8.9%)	146 (9.3%)	45 (8.7%)	166 (6.5%)
	0─9	42 (5.0%)	149 (3.6%)	18 (5.7%)	62 (3.9%)	24 (4.6%)	87 (3.4%)
	Missing	230 (27.6%)	935 (22.7%)	73 (23.1%)	344 (21.9%)	157 (30.3%)	591 (23.2%)
Costs [3]	Mean (SD)	3440 (8007)	3546 (7423)	3526 (6738)	3210 (5838)	3387 (8699)	3753 (8247)
Any dental procedure [4]	N	501 (60.6%)	2808 (68.5%)	212 (67.5%)	1074 (68.5%)	289 (56.3%)	1734 (68.5%)

[1] Comprises patients who were diagnosed with H&N cancer during 2010─2012, had recorded data in the time period 2009─2013 and recorded data for the time frame ±2 years of the year of cancer diagnosis, and the unexposed cohort.[2] All characteristics determined at baseline the year before the year of cancer diagnosis.
[3] Total annual costs for dental care utilization (SEK 100 is approximately EUR 10).
[4] Number of subjects with at least one dental procedure on record.
Unexpo = Unexposed; Non-irrad = Non-irradiated; Irrad = Irradiated; SD = standard deviation
