# Supplementary material for: Consumption and direct costs of dental care for patients with head and neck cancer: A 16-year cohort study
Source: PLoS One. 2017 Aug 23;12(8):e0182877. doi: 10.1371/journal.pone.0182877 (PMC5568378; doi:10.1371/journal.pone.0182877)
Supplement: S2 Table — 1 Comprises patients who were diagnosed with H&N cancer during 2000–2007 and had recorded data in the time period 2009–2013, and the matched population without H&N cancer. 2 All characteristics determined at baseline, the year before the year of cancer diagnosis. (PDF) [file pone.0182877.s002.pdf]

S2 Characteristics of the exposed and unexposed cohorts in the long-term follow-up. [1]

Characteristic [2]	Description	Exposed cohort	Unexposed cohort	Exposed: Subgroup Non-irrad	Unexposed matched to Non-irrad	Exposed: Subgroup Irrad	Unexposed matched to Irrad
Total	N	1032 (100.0%)	4622 (100.0%)	640 (100.0%)	2860 (100.0%)	392 (100.0%)	1762 (100.0%)
Sex	Male	623 (60.4%)	2765 (59.8%)	369 (57.7%)	1637 (57.2%)	254 (64.8%)	1128 (64.0%)
	Female	409 (39.6%)	1857 (40.2%)	271 (42.3%)	1223 (42.8%)	138 (35.2%)	634 (36.0%)
Age (years)	Mean (SD)	61.7 (13.5)	60.6 (13.1)	62.7 (14.0)	61.4 (13.6)	60.0 (12.5)	59.1 (12.1)
	<20	9 (0.9%)	43 (0.9%)	8 (1.3%)	38 (1.3%)	1 (0.3%)	5 (0.3%)
	20 – <60	433 (42.0%)	2089 (45.2%)	249 (38.9%)	1215 (42.5%)	184 (46.9%)	874 (49.6%)
	60 – <80	496 (48.1%)	2189 (47.4%)	311 (48.6%)	1382 (48.3%)	185 (47.2%)	807 (45.8%)
	80 +	94 (9.1%)	301 (6.5%)	72 (11.3%)	225 (7.9%)	22 (5.6%)	76 (4.3%)
Education	Missing	24 (2.3%)	107 (2.3%)	19 (3.0%)	82 (2.9%)	5 (1.3%)	25 (1.4%)
	Primary	299 (29.0%)	1147 (24.8%)	191 (29.8%)	756 (26.4%)	108 (27.6%)	391 (22.2%)
	Secondary	421 (40.8%)	1905 (41.2%)	256 (40.0%)	1124 (39.3%)	165 (42.1%)	781 (44.3%)
	University	288 (27.9%)	1463 (31.7%)	174 (27.2%)	898 (31.4%)	114 (29.1%)	565 (32.1%)
Family income	Missing	7 (0.7%)	35 (0.8%)	7 (1.1%)	34 (1.2%)		1 (0.1%)
	Lower 3rd	382 (37.0%)	1608 (34.8%)	237 (37.0%)	1017 (35.6%)	145 (37.0%)	591 (33.5%)
	Middle 3rd	372 (36.0%)	1556 (33.7%)	230 (35.9%)	925 (32.3%)	142 (36.2%)	631 (35.8%)
	Upper 3rd	271 (26.3%)	1423 (30.8%)	166 (25.9%)	884 (30.9%)	105 (26.8%)	539 (30.6%)

[1] Comprises patients who were diagnosed with H&N cancer during 2000−2007, had recorded data in the time period 2009−2013, and the unexposed cohort.
[2] All characteristics determined at baseline, the year before the year of cancer diagnosis.
Non-irrad = Non-irradiated; Irrad = Irradiation; SD = standard deviation
