# Supplementary material for: Consumption and direct costs of dental care for patients with head and neck cancer: A 16-year cohort study
Source: PLoS One. 2017 Aug 23;12(8):e0182877. doi: 10.1371/journal.pone.0182877 (PMC5568378; doi:10.1371/journal.pone.0182877)
Supplement: S3 Table — (PDF) [file pone.0182877.s003.pdf]

S3 Short-term follow-up: Costs before and after cancer diagnosis in the exposed and unexposed cohorts - Unadjusted analysis.

Variable	Year relative diagnosis	Exposed cohort		Unexposed cohort	Pairwise comparisons (*p*-value)		
		Non-irradiated	Irradiated				
		LSMeans (95% CI)	LSMeans (95% CI)	LSMeans (95% CI)	Non-irrad vs Unexpo	Irrad vs Unexpo	Non-irrad vs Rad
Direct costs to patient	-2	2585 (2078-3093)	1949 (1528-2371)	2156 (2012-2301)	.11	.36	.059
	-1	2270 (1839-2702)	1970 (1636-2303)	2149 (2031-2267)	.59	.32	.28
	0	2066 (1635-2497)	1637 (1304-1970)	2150 (2032-2268)	.71	.0044	.12
	1	2226 (1774-2678)	1713 (1370-2056)	2171 (2048-2294)	.82	.014	.076
	2	2162 (1548-2777)	2131 (1673-2589)	2222 (2055-2388)	.86	.72	.94
Total costs	-2	4210 (3061-5359)	3121 (2161-4080)	3505 (3177-3834)	.25	.46	.15
	-1	3602 (2636-4567)	3445 (2698-4193)	3535 (3271-3799)	.90	.82	.80
	0	6042 (5078-7006)	12424 (11679-13170)	3556 (3293-3820)	<.0001	<.0001	<.0001
	1	7414 (6402-8426)	10275 (9506-11045)	3614 (3338-3890)	<.0001	<.0001	<.0001
	2	5282 (3880-6684)	5872 (4828-6916)	3774 (3393-4154)	.042	.0002	.51

Unexpo = Unexposed; Non-irrad = Non-irradiated; Irrad = Irradiated; LSMeans = Least-squares means
