# Supplementary material for: Consumption and direct costs of dental care for patients with head and neck cancer: A 16-year cohort study
Source: PLoS One. 2017 Aug 23;12(8):e0182877. doi: 10.1371/journal.pone.0182877 (PMC5568378; doi:10.1371/journal.pone.0182877)
Supplement: S4 Table — (PDF) [file pone.0182877.s004.pdf]

S4 Short-term follow-up: Number of procedures before and after cancer diagnosis in the exposed and unexposed cohorts (ANCOVA model) - Unadjusted analysis.

Variable	Year relative diagnosis	Exposed subgroup: Non-irradiated LSMeans (95% CI)	Exposed subgroup: Irradiated LSMeans (95% CI)	Unexposed cohort LSMeans (95% CI)	Pairwise comparisons (*p*-value): Non-irrad vs Unexpo	Irrad vs Unexpo	Non-irrad vs Irrad
All	-2	3.96 (3.38-4.55)	3.27 (2.78-3.75)	3.53 (3.37-3.70)	.17	.30	.072
	-1	3.58 (3.07-4.09)	3.12 (2.73-3.51)	3.54 (3.40-3.68)	.90	.047	.16
	0	5.44 (4.93-5.94)	8.28 (7.89-8.67)	3.52 (3.38-3.66)	<.0001	<.0001	<.0001
	1	5.51 (4.99-6.04)	7.53 (7.12-7.93)	3.40 (3.26-3.54)	<.0001	<.0001	<.0001
	2	4.44 (3.74-5.14)	4.27 (3.75-4.80)	3.54 (3.35-3.73)	.014	.0091	.71
Examination	-2	1.32 (1.10-1.54)	1.20 (1.01-1.38)	1.36 (1.29-1.42)	.75	.10	.40
	-1	1.43 (1.24-1.63)	1.16 (1.01-1.31)	1.38 (1.33-1.43)	.62	.0067	.031
	0	2.37 (2.17-2.56)	4.06 (3.91-4.21)	1.40 (1.34-1.45)	<.0001	<.0001	<.0001
	1	2.18 (1.98-2.38)	3.06 (2.91-3.22)	1.38 (1.33-1.44)	<.0001	<.0001	<.0001
	2	1.75 (1.49-2.02)	1.60 (1.40-1.80)	1.47 (1.40-1.54)	.042	.22	.36
Preventive and supportive	-2	0.99 (0.82-1.16)	0.86 (0.72-1.00)	0.91 (0.86-0.96)	.37	.50	.24
	-1	0.97 (0.81-1.12)	0.80 (0.68-0.91)	0.91 (0.87-0.96)	.52	.064	.085
	0	1.53 (1.38-1.69)	2.60 (2.48-2.72)	0.93 (0.89-0.97)	<.0001	<.0001	<.0001
	1	1.84 (1.68-2.00)	2.78 (2.66-2.90)	0.90 (0.86-0.94)	<.0001	<.0001	<.0001
	2	1.28 (1.08-1.49)	1.31 (1.16-1.46)	0.91 (0.86-0.97)	.0006	<.0001	.82
Surgical	-2	0.17 (0.08-0.26)	0.20 (0.12-0.28)	0.17 (0.14-0.19)	.94	.38	.59
	-1	0.15 (0.08-0.23)	0.26 (0.20-0.32)	0.16 (0.14-0.18)	.83	.0025	.032
	0	0.29 (0.21-0.36)	0.44 (0.38-0.50)	0.14 (0.12-0.17)	.0006	<.0001	.0028
	1	0.17 (0.09-0.25)	0.21 (0.15-0.28)	0.13 (0.11-0.16)	.36	.017	.44
	2	0.13 (0.01-0.24)	0.22 (0.14-0.31)	0.13 (0.10-0.16)	.99	.043	.19
Endodontic	-2	0.07 (0.02-0.13)	0.03 (-.01-0.08)	0.08 (0.06-0.09)	.82	.074	.30
	-1	0.04 (-.00-0.09)	0.07 (0.03-0.10)	0.07 (0.06-0.09)	.17	.70	.39
	0	0.05 (0.01-0.10)	0.08 (0.04-0.11)	0.08 (0.07-0.09)	.21	.87	.35
	1	0.05 (-.00-0.09)	0.07 (0.03-0.10)	0.07 (0.06-0.08)	.38	.93	.51
	2	0.12 (0.05-0.19)	0.06 (0.01-0.10)	0.08 (0.06-0.10)	.29	.33	.13
Restorative	-2	0.90 (0.73-1.08)	0.60 (0.46-0.74)	0.66 (0.61-0.71)	.0078	.42	.0075
	-1	0.62 (0.47-0.77)	0.53 (0.41-0.64)	0.68 (0.64-0.72)	.48	.019	.35
	0	0.75 (0.60-0.90)	0.44 (0.33-0.55)	0.65 (0.61-0.70)	.21	.0005	.0011
	1	0.61 (0.46-0.77)	0.48 (0.37-0.60)	0.60 (0.56-0.64)	.87	.072	.19
	2	0.62 (0.42-0.83)	0.63 (0.47-0.78)	0.61 (0.56-0.67)	.92	.87	.98
Prosthodontic	-2	0.48 (0.29-0.68)	0.36 (0.19-0.52)	0.34 (0.29-0.40)	.18	.85	.35
	-1	0.34 (0.17-0.50)	0.29 (0.17-0.42)	0.32 (0.28-0.36)	.86	.70	.69
	0	0.39 (0.23-0.55)	0.55 (0.43-0.68)	0.29 (0.24-0.33)	.23	.0001	.12
	1	0.57 (0.40-0.74)	0.76 (0.63-0.89)	0.30 (0.25-0.35)	.0028	<.0001	.083
	2	0.46 (0.22-0.70)	0.37 (0.20-0.55)	0.30 (0.24-0.37)	.21	.47	.56

Unexpo = Unexposed; Non-irrad = Non-irradiated; Irrad = Irradiated; LSMeans = Least-squares means
