# Supplementary material for: Consumption and direct costs of dental care for patients with head and neck cancer: A 16-year cohort study
Source: PLoS One. 2017 Aug 23;12(8):e0182877. doi: 10.1371/journal.pone.0182877 (PMC5568378; doi:10.1371/journal.pone.0182877)
Supplement: S5 Table — (PDF) [file pone.0182877.s005.pdf]

S5 Long-term follow-up: Costs before and after cancer diagnosis in the exposed and unexposed cohorts - Unadjusted analysis.

Variable	Exposed cohort		Unexposed cohort	Pairwise comparisons (*p*-value)		
	Non-irradiated	Irradiated				
	LSMeans (95% CI)	LSMeans (95% CI)	LSMeans (95% CI)	Non-irrad vs Unexpo	Irrad vs Unexpo	Non-irrad vs Irrad
Direct costs to patient	2036 (1835-2238)	1742 (1476-2007)	2107 (2034-2181)	.52	.0093	.083
Total costs	5544 (5092-5996)	5685 (5089-6280)	3535 (3370-3700)	<.0001	<.0001	.71

Unexpo = Unexposed; Non-irrad = Non-irradiated; Irrad = Irradiated; LSMeans = Least-squares means
