# Supplementary material for: Consumption and direct costs of dental care for patients with head and neck cancer: A 16-year cohort study
Source: PLoS One. 2017 Aug 23;12(8):e0182877. doi: 10.1371/journal.pone.0182877 (PMC5568378; doi:10.1371/journal.pone.0182877)
Supplement: S6 Table — (PDF) [file pone.0182877.s006.pdf]

S6 Long-term follow-up: Number of procedures before and after cancer diagnosis in the exposed and unexposed cohorts (ANCOVA model) - Unadjusted analysis.

Variable	Exposed cohort		Unexposed cohort	Pairwise comparisons (p-value)		
	Non-irradiated	Irradiated				
	LSMeans (95% CI)	LSMeans (95% CI)	LSMeans (95% CI)	Non-irrad vs Unexpo	Irrad vs Unexpo	Non-irrad vs Irrad
All	4.14 (3.87-4.41)	4.12 (3.76-4.47)	3.40 (3.31-3.50)	<.0001	.0002	.93
Examination	1.59 (1.49-1.69)	1.50 (1.37-1.64)	1.35 (1.31-1.39)	<.0001	.033	.33
Preventive and supportive	1.03 (0.95-1.11)	1.08 (0.97-1.19)	0.89 (0.86-0.92)	.0030	.0013	.45
Surgical	0.22 (0.19-0.25)	0.21 (0.17-0.25)	0.15 (0.13-0.16)	<.0001	.0038	.71
Endodontic	0.07 (0.05-0.09)	0.07 (0.04-0.09)	0.08 (0.07-0.08)	.67	.54	.82
Restorative	0.65 (0.57-0.73)	0.74 (0.64-0.85)	0.61 (0.58-0.64)	.43	.017	.14
Prosthodontic	0.54 (0.47-0.61)	0.44 (0.35-0.54)	0.31 (0.29-0.34)	<.0001	0.0075	.100

Unexpo = Unexposed; Non-irrad = Non-irradiated; Irrad = Irradiated; LSMeans = Least-squares means
